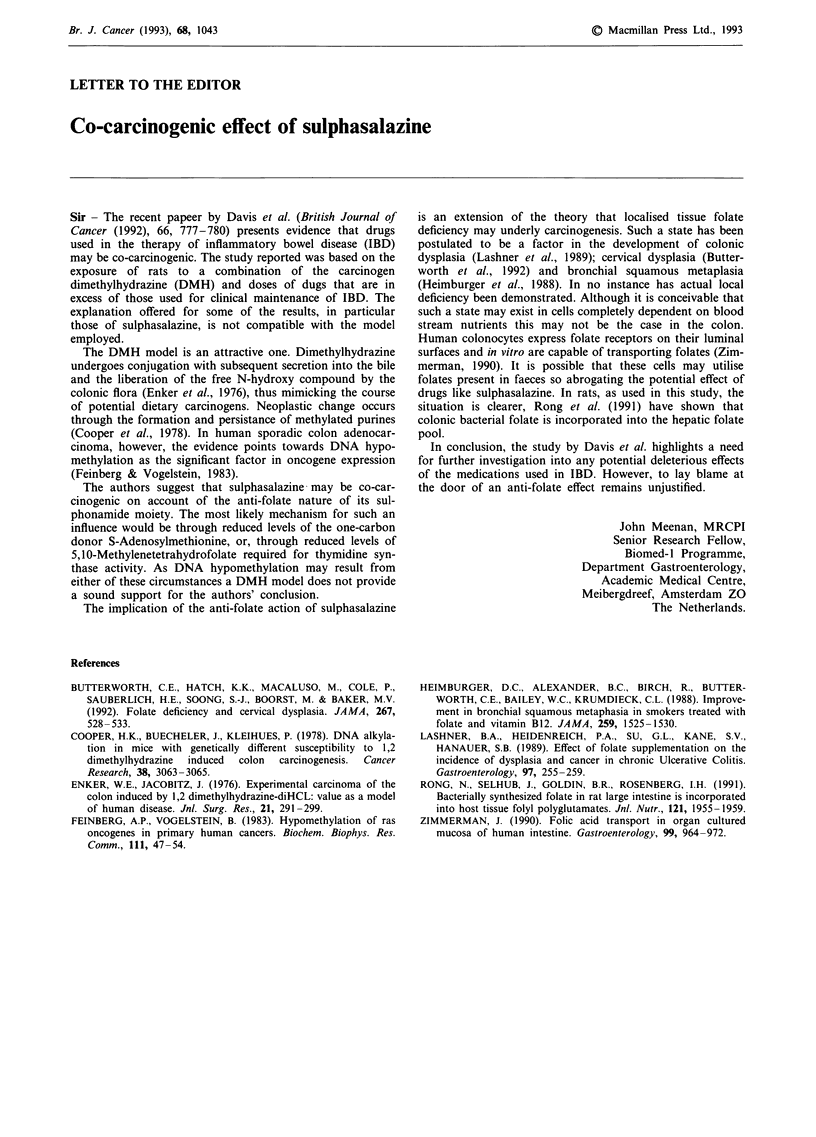# Co-carcinogenic effect of sulphasalazine.

**DOI:** 10.1038/bjc.1993.477

**Published:** 1993-11

**Authors:** J. Meenan


 © Macmillan Press Ltd., 1993

### LETTER TO THE EDITOR

### Co-carcinogenic effect of sulphasalazine

**Sir** – The recent papeer by Davis *et al.* (*British Journal of Cancer* (1992), 66, 777–780) presents evidence that drugs used in the therapy of inflammatory bowel disease (IBD) may be co-carcinogenic. The study reported was based on the exposure of rats to a combination of the carcinogen dimethylhydrazine (DMH) and doses of dugs that are in excess of those used for clinical maintenance of IBD. The explanation offered for some of the results, in particular those of sulphasalazine, is not compatible with the model employed.

The DMH model is an attractive one. Dimethylhydrazine undergoes conjugation with subsequent secretion into the bile and the liberation of the free N-hydroxy compound by the colonic flora (Enker *et al.*, 1976), thus mimicking the course of potential dietary carcinogens. Neoplastic change occurs through the formation and persistance of methylated purines (Cooper *et al.*, 1978). In human sporadic colon adenocarcinoma, however, the evidence points towards DNA hypomethylation as the significant factor in oncogene expression (Feinberg & Vogelstein, 1983).

The authors suggest that sulphasalazine may be co-carcinogenic on account of the anti-folate nature of its sulphonamide moiety. The most likely mechanism for such an influence would be through reduced levels of the one-carbon donor S-Adenosylmethionine, or, through reduced levels of 5,10-Methylenetetrahydrofolate required for thymidine synthase activity. As DNA hypomethylation may result from either of these circumstances a DMH model does not provide a sound support for the authors' conclusion.

The implication of the anti-folate action of sulphasalazine is an extension of the theory that localised tissue folate deficiency may underly carcinogenesis. Such a state has been postulated to be a factor in the development of colonic dysplasia (Lashner *et al.*, 1989); cervical dysplasia (Butterworth *et al.*, 1992) and bronchial squamous metaplasia (Heimburger *et al.*, 1988). In no instance has actual local deficiency been demonstrated. Although it is conceivable that such a state may exist in cells completely dependent on blood stream nutrients this may not be the case in the colon. Human colonocytes express folate receptors on their luminal surfaces and *in vitro* are capable of transporting folates (Zimmerman, 1990). It is possible that these cells may utilise folates present in faeces so abrogating the potential effect of drugs like sulphasalazine. In rats, as used in this study, the situation is clearer, Rong *et al.* (1991) have shown that colonic bacterial folate is incorporated into the hepatic folate pool.

In conclusion, the study by Davis *et al.* highlights a need for further investigation into any potential deleterious effects of the medications used in IBD. However, to lay blame at the door of an anti-folate effect remains unjustified.

John Meenan, MRCPI
Senior Research Fellow,
Biomed-1 Programme,
Department Gastroenterology,
Academic Medical Centre,
Meibergdreef, Amsterdam ZO
The Netherlands.

### References

BUTTERWORTH, C.E., HATCH, K.K., MACALUSO, M., COLE, P., SAUBERLICH, H.E., SOONG, S.-J., BOORST, M. & BAKER, M.V. (1992). Folate deficiency and cervical dysplasia. *JAMA*, **267**, 528–533.

COOPER, H.K., BUECHELER, J., KLEIHUES, P. (1978). DNA alkylation in mice with genetically different susceptibility to 1,2 dimethylhydrazine induced colon carcinogenesis. *Cancer Research*, **38**, 3063–3065.

ENKER, W.E., JACOBITZ, J. (1976). Experimental carcinoma of the colon induced by 1,2 dimethylhydrazine-diHCL: value as a model of human disease. *Jnl. Surg. Res.*, **21**, 291–299.

FEINBERG, A.P., VOGELSTEIN, B. (1983). Hypomethylation of ras oncogenes in primary human cancers. *Biochem. Biophys. Res. Comm.*, **111**, 47–54.

HEIMBURGER, D.C., ALEXANDER, B.C., BIRCH, R., BUTTERWORTH, C.E., BAILEY, W.C., KRUMDIECK, C.L. (1988). Improvement in bronchial squamous metaphasia in smokers treated with folate and vitamin B12. *JAMA*, **259**, 1525–1530.

LASHNER, B.A., HEIDENREICH, P.A., SU, G.L., KANE, S.V., HANAUER, S.B. (1989). Effect of folate supplementation on the incidence of dysplasia and cancer in chronic Ulcerative Colitis. *Gastroenterology*, **97**, 255–259.

RONG, N., SELHUB, J., GOLDIN, B.R., ROSENBERG, I.H. (1991). Bacterially synthesized folate in rat large intestine is incorporated into host tissue folyl polyglutamates. *Jnl. Nutr.*, **121**, 1955–1959.

ZIMMERMAN, J. (1990). Folic acid transport in organ cultured mucosa of human intestine. *Gastroenterology*, **99**, 964–972.